# Acute Native Aortic Valve Thrombosis in the Setting of Extracorporeal Membrane Oxygenation

**DOI:** 10.7759/cureus.78561

**Published:** 2025-02-05

**Authors:** Loruanma Lam, Julien Feghaly, Emil Missov

**Affiliations:** 1 Internal Medicine, University of Florida College of Medicine – Jacksonville, Jacksonville, USA; 2 Cardiology, University of Florida College of Medicine – Jacksonville, Jacksonville, USA

**Keywords:** aortic valve, aortic valve repair, aortic valve thrombus, extracorporeal membrane oxygenation complication, extracorporeal membrane oxygenation support

## Abstract

Aortic valve (AV) thrombosis is a rare but clinically significant condition with fewer than 80 cases of native AV thrombosis reported over the past 50 years. The clinical presentation varies widely, ranging from asymptomatic cases to acute myocardial infarction (MI), with or without cardiogenic shock. MI has been identified as the most common presentation and hypercoagulability as the most prevalent underlying etiology. Extracorporeal membrane oxygenation (ECMO) is a rare but documented cause of AV thrombosis due to the potential for clot formation in the setting of insufficient left ventricle (LV) offloading and decreased LV function. People with inherited thrombophilias and those on ECMO have an increased risk of developing thrombosis but via different mechanisms and in different settings. Diseases of hypercoagulability predispose the patient to venous thromboembolism (VTE) due to intrinsic blood abnormalities. Patients on ECMO face a risk of thrombosis due to mechanical challenges that can arise from the ECMO circuit itself and systemic inflammation. Transesophageal echocardiography (TEE) is the diagnostic modality of choice, which offers the highest sensitivity for detection of AV thrombus compared to cardiac computed tomography angiography or cardiac magnetic resonance imaging.

While many interventions exist for the management of AV thrombosis, there are no current guidelines that outline the best treatment approach. Here we present a case of native AV thrombosis that developed in a patient on ECMO following an elective left heart catheterization (LHC). The patient suffered a series of complications that led to her requiring central veno-arterial (VA) ECMO when unable to be weaned from cardiac bypass. During evaluation for ECMO turndown via TEE guidance, the patient was found to have a large AV valve thrombus. Given the patient’s extremely poor clinical prognosis, the decision was made to pursue a compassionate wean. Current interventions include anticoagulation, surgical thrombectomy, and systemic thrombolytics. However, a lack of randomized control trials outlining the best treatment approach remain unclear. While it may not be feasible given the critical nature of its presentation and rarity, further research is needed to guide the management of native AV thrombosis.

## Introduction

Aortic valve (AV) thrombosis is a rare but clinically significant condition with fewer than 80 cases of native AV thrombosis documented in the literature in the past 50 years. It is a clinically difficult condition to manage given the lack of guidelines outlining the best treatment approach. Additionally, it is a source for embolic events with possible adverse outcomes. A meta-analysis by Alajaji et al. identified an underlying hypercoagulable state as the most common etiology, followed by AV abnormalities [1]. Myocardial infarction (MI) was identified as the most common presentation [1,2]. Other presentations include stroke, acute AV insufficiency (with or without cardiogenic shock) or even asymptomatic [2,3]. Echocardiography is the recommended imaging modality, while transesophageal echocardiography (TEE) holds the highest sensitivity due to proximity, resolution advantage, and ability to directly visualize the thrombus [4-6]. In combination with aortic root angiography, the sensitivity approaches 100%. Cardiac CT angiography (CTA) offers a sensitivity of about 86% and cardiac MRI is highly sensitive in detecting intra-cardiac thrombi. However, both require transportation to imaging, which is often not feasible given that most of the patients in this population are critically ill and unstable. The risk of AV thrombosis while on extracorporeal membrane oxygenation (ECMO), specifically veno-arterial (VA) ECMO, is mostly due to decreased left ventricular unloading in combination with blood stasis in the aortic root when the left ventricle (LV) ejection fraction (EF) is depressed [6]. To reduce these potential scenarios, interventions such as percutaneous LV venting or mechanical support can be introduced, i.e., intra-aortic balloon pump insertion. 

Current guidelines emphasize the importance of frequent monitoring of LV decompression and opening of the AV [7]. Regarding the management of AV thrombosis itself, current interventions include anticoagulation, thrombolytic therapy, and/or surgical valve replacement or thrombectomy. The decision on which intervention to pursue depends on several factors such as the patient’s age, bleeding risk, candidacy for surgery and overall prognosis. Furthermore, a lack of randomized studies has left an optimal treatment regimen unclear, e.g., pursuing early surgical thrombectomy with anticoagulation versus anticoagulation alone [8]. Nonetheless, timely recognition remains an integral step in the management that follows for this patient population. Here we present a case of native AV thrombosis that developed in a patient on central VA ECMO following an elective left heart catheterization (LHC) and AV repair.

## Case presentation

**Figure 1:**
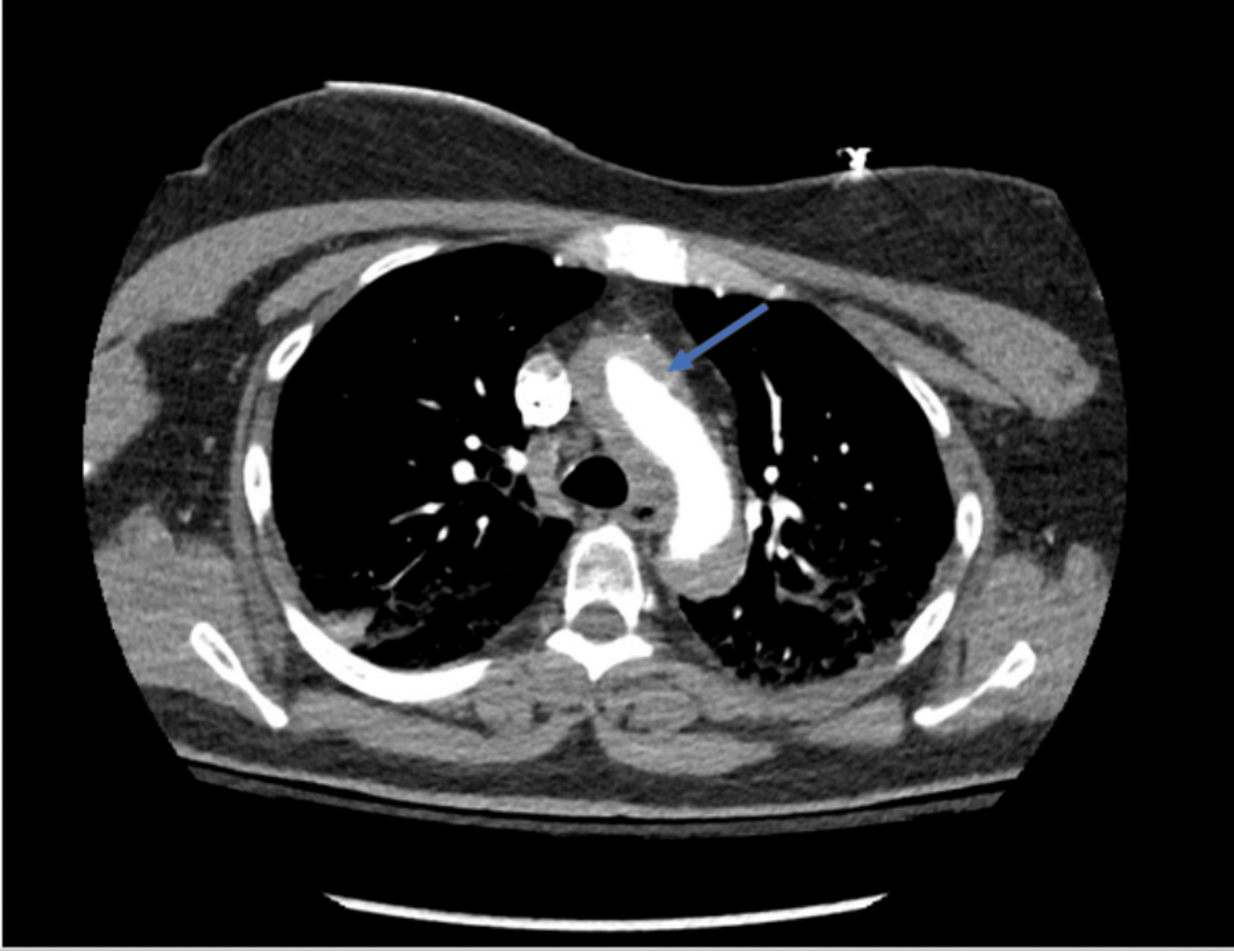
CTA of the chest (axial view) with and without IV contrast showing acute type A Stanford dissection (arrow) involving the ascending and descending aorta CTA: CT angiography

**Figure 2:**
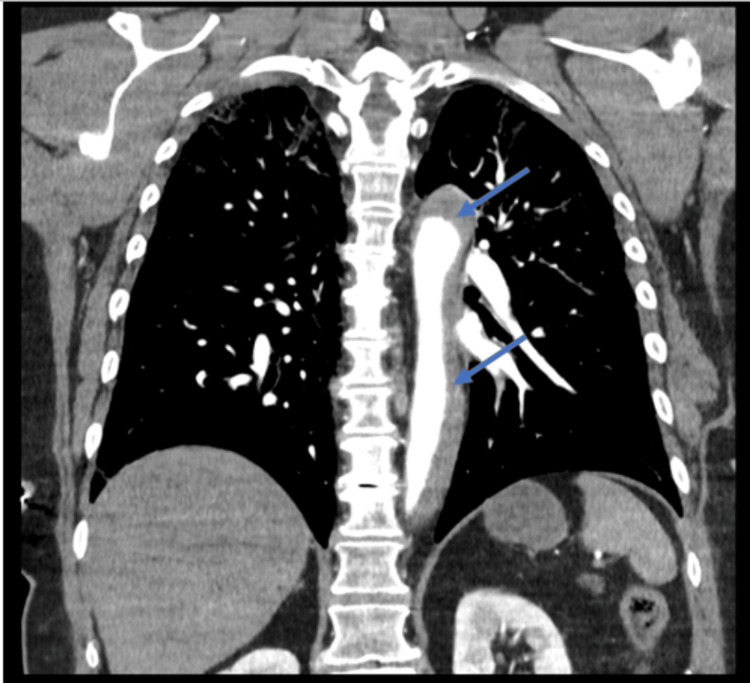
CTA of the chest (coronal view) with and without IV contrast showing acute type A Stanford dissection (arrows) involving the ascending aorta with significant extension into the descending thoracic aorta CTA: CT angiography

**Figure 3:**
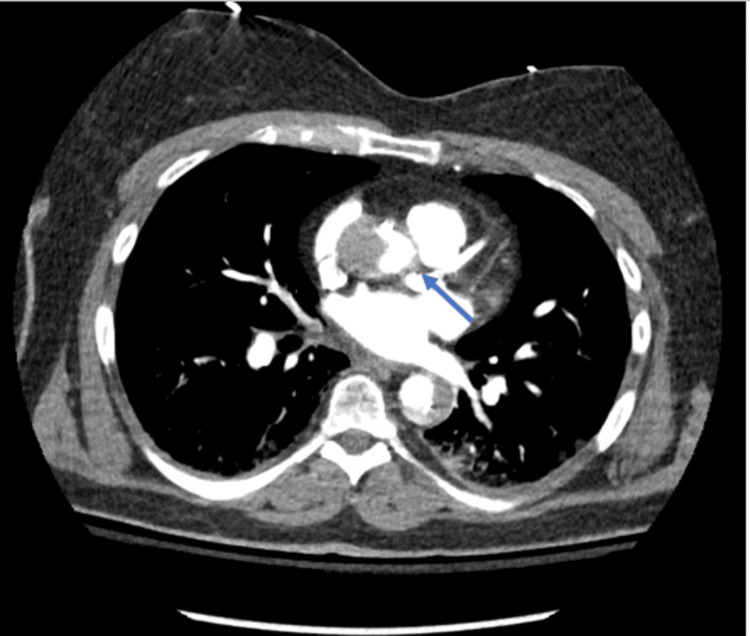
CTA chest with and without IV contrast showing acute type A Stanford dissection that abuts (arrow) the LMCA CTA: CT angiography; LMCA: Left main coronary artery

A 64-year-old female with a medical history of hypertension, hyperlipidemia, and former tobacco use (40-pack-year) initially presented to Florida Memorial Hospital for an elective LHC. She was fully independent at baseline and did light exercise two times a week with mild shortness of breath, without symptoms of angina. She had no known cardiac history including coronary artery disease, MI, arrhythmias, or congestive heart failure. The LHC revealed greater than 70% disease of the distal left anterior descending (LAD) artery. The procedure was complicated by a left main coronary artery (LMCA) dissection extending into the LAD and left circumflex (LCX) arteries that occurred immediately after a bout of coughing on the table. She successfully underwent immediate stenting (using drug-eluting stents) of the LMCA, LAD, and LCX as management of the dissection. She developed chest pain and hypotension which prompted an intra-operative TEE, which revealed a reduced EF of 25% and aortic dissection. CTA of the chest showed a Stanford type A aortic dissection (Figures 1, 2) extending from the AV plane into the suprarenal abdominal aorta, with the dissection flap approximating the origin of the right coronary artery and ostial LMCA (Figure 3).

An Impella cardiac power (CP) left ventricular assist device was placed for the management of cardiogenic shock. Upon adjustment of the device, she suffered cardiac arrest, thought to be due to low volume in the setting of her acute dissection. After the return of spontaneous circulation, the patient was transferred to our hospital for cardiothoracic surgery repair of the acute dissection via the Bentall procedure as it was thought to be the cause of her cardiac arrest. She also underwent coronary artery bypass grafting (CABG) using sequential saphenous vein grafting to the ramus and distal LAD artery. She was unable to come off cardiac bypass post procedure due to left ventricular hypokinesis seen on intra-operative TEE. Her blood pressure remained low, and laboratory results revealed a worsening metabolic acidosis and rising lactic acid. Hemoglobin remained between 9 g/dL and 10 g/dL. She was placed on vasoactive support with norepinephrine and dobutamine with minimal improvement in her hemodynamics, necessitating the initiation of central VA ECMO. Her blood pressure improved and repeated laboratory studies showed a down-trending serum lactic acid as well as improvement in arterial oxygen saturation on arterial blood gas. She remained on flow rates between 3.5 and 4.3L/min. Direct LV unloading was provided via additional cannula insertion into the LV apex.

**Figure 4:**
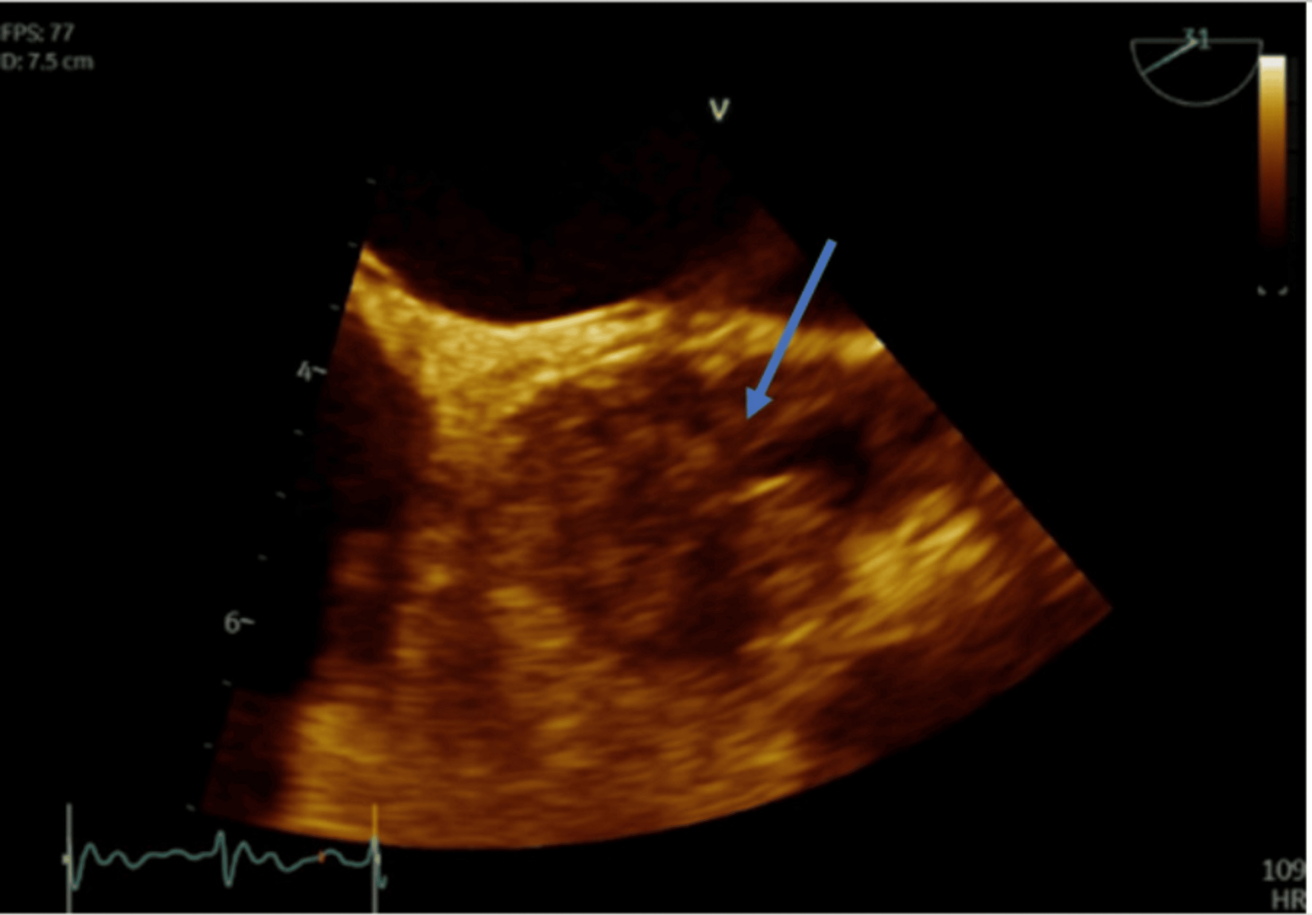
TEE, mid-esophageal short-axis view showing thrombus (arrow) of the AV along all three coronary cusps extending into the LMCA ostium TEE: Transesophageal echocardiogram; AV: Aortic valve; LMCA: Left main coronary artery

**Figure 5:**
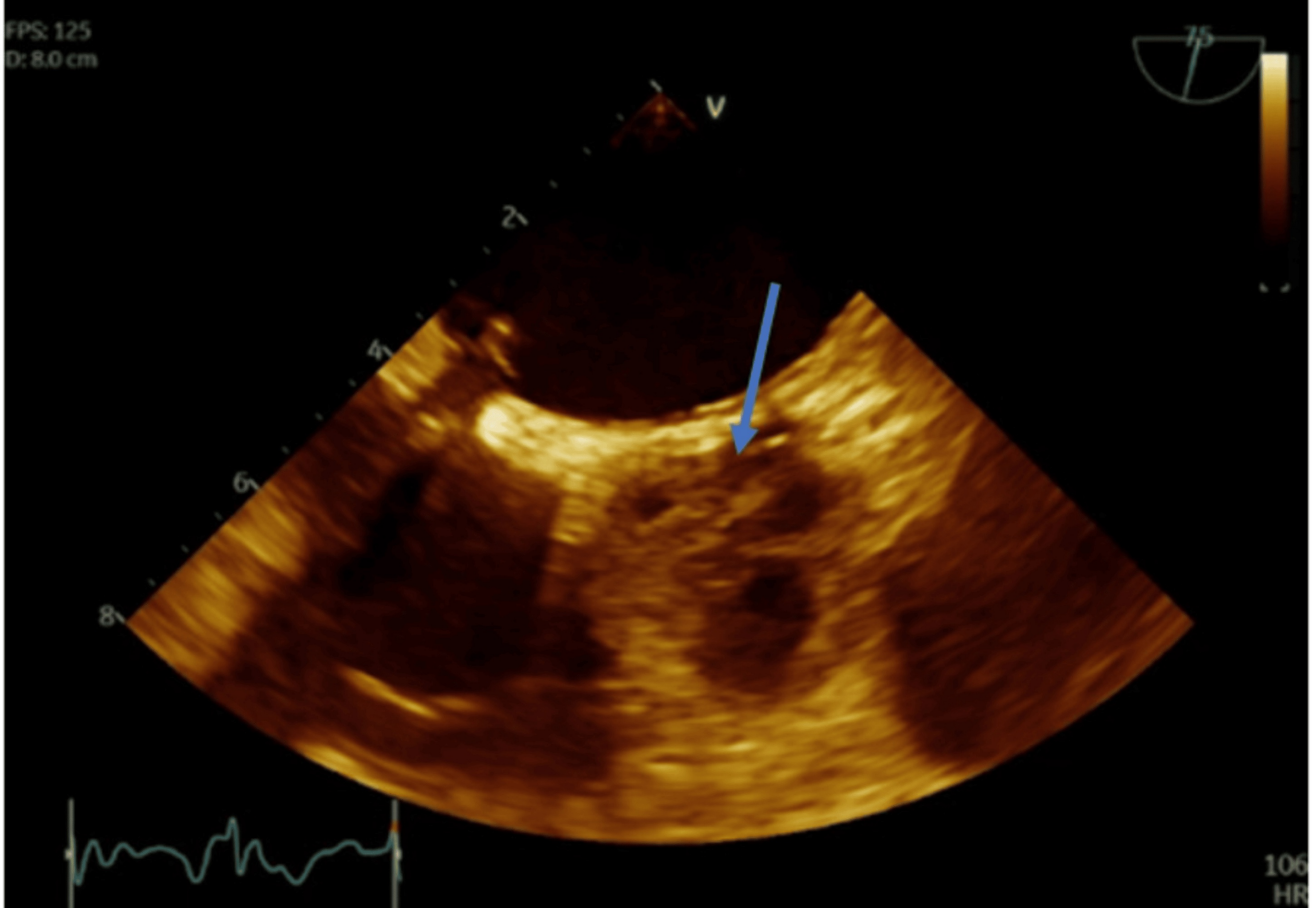
TEE, mid-esophageal short-axis view, zoomed out, showing thrombus of the AV (arrow) TEE: Transesophageal echocardiogram; AV: Aortic valve

On postoperative day 3, during evaluation for ECMO turn-down under TEE guidance, the AV and sinuses were found to be completely thrombosed extending into the LMCA and RCA (Figures 4, 5, Video 1). Upon review of the activated partial thromboplastin time (aPTT) levels during unfractionated heparin infusion, the patient remained at therapeutic levels for central VA ECMO (50-70 seconds) from the introduction of the circuit to postoperative day 3 when the AV thrombus was discovered. Given the extremely poor prognosis and critical illness, a decision was made for a compassionate wean. The patient passed away within minutes of stopping ECMO.

**Video 1:** AV thrombosis (observed echogenicity causing poor leaflet mobility) with extension of the thrombus into the LMCA AV: Aortic valve; LMCA: Left main coronary artery

## Discussion

This case presents an unusual occurrence of AV thrombosis. Native heart valve thrombosis is a rare yet underestimated valvular pathology. Despite hypercoagulable disease being the most common etiology and MI the most common presentation, this patient had no known underlying hypercoagulable disease and no electrocardiogram (ECG) evidence of acute ischemia suggestive of MI. A thrombophilia workup was not able to be done however due to the patient's critical condition. During her aortic dissection repair, she underwent two-vessel bypass surgery with grafting and re-suspension of her AV. The valve appeared intact, with no signs of sinus disruption, and the coronary artery ostium was patent. No thrombus was visualized in the surgical field. Due to persistent high inotropic support requirements when attempting to wean from cardiac bypass, central VA ECMO was implemented. Patients on ECMO face a risk of thrombosis due to mechanical challenges that can arise from the ECMO circuit itself. There is a potential for clot formation in the setting of insufficient LV offloading and decreased LV function, as well as retrograde blood flow in the aortic root. To reduce these potential scenarios, interventions such as percutaneous LV venting or mechanical support can be introduced, i.e., intra-aortic balloon pump insertion. 

As mentioned previously, the AV and sinuses were found to be completely thrombosed during evaluation for ECMO turn-down under TEE guidance. Is it unclear how often visualization via TEE was done, and it raises the question whether more frequent monitoring would have caught early clot formation. She was on therapeutic levels of heparin while on ECMO but was not on aspirin or other antiplatelet drug. While aortic thrombus may occur with ECMO despite LV unloading, it is more likely to occur with higher ECMO flows, which were not present in this case. Another point to make is the higher thrombotic risk associated with peripheral ECMO versus central VA ECMO. Several factors likely played a role in the development of this patient’s AV thrombus, with inflammation from direct tissue manipulation playing a major role. Through the activation of inflammatory cytokines from her coronary artery dissections requiring stenting, aortic dissection repair, and AV repair, it potentially set the stage for endothelial injury and impaired anticoagulant pathways, ultimately leading to AV thrombosis. While the different interventions for thrombus removal were not reached in this case due to the hospice route that was pursued, it is still crucial to discuss. Anticoagulation remains the cornerstone of treatment. In this patient, it is difficult to determine whether more aggressive anticoagulation strategies at the beginning of ECMO implementation would have reduced the risk of clot formation. Surgical or catheter associated thrombectomy could have served as adjunctive therapies for better clot removal with a lower risk of embolization. While the meta-analysis by Alajaji et al. found that some case reports had better outcomes when anticoagulation was used in combination with mechanical clot retrieval, the more robust data regarding optimal management remains very limited [1].

## Conclusions

Native AV thrombosis remains an uncommon clinical diagnosis that is challenging to treat, with an in-hospital mortality risk estimated at 20%. For prosthetic valve thrombosis, the mortality varies depending on the timing of thrombus formation and type of prosthesis used. Optimal medical treatment remains uncertain due to limited studies and a lack of randomized trials. Current treatment options include anticoagulation, surgical thrombectomy, and/or thrombolysis. Antithrombotic management can be complex, and limited data shows that surgical thrombectomy may carry better outcomes than antithrombotic therapy alone. Furthermore, surgical thrombectomy may offer a benefit over catheter-associated thrombectomy due to more thrombus removal and lower risk of embolization. This case underscores the critical role of TEE in identifying AV thrombosis and also highlights the importance of appropriate anticoagulation during mechanical support in the setting of critical illness, specifically with high-dose, low infusion heparin. Increased awareness of this phenomenon is crucial in patients who present with MI and embolic events with non-revealing coronary angiography. Even looking beyond optimal management, future randomized trials are needed to investigate the frequency of monitoring with imaging in addition to directions for earlier interventions for LV offloading in an attempt to further decrease the risk of native AV thrombosis.
